# Comprehensive phylogenomic analysis of Zika virus: Insights into its origin, past evolutionary dynamics, and global spread

**DOI:** 10.1016/j.virusres.2024.199490

**Published:** 2024-11-08

**Authors:** Nicola Zadra, Annapaola Rizzoli

**Affiliations:** a Conservation Genomics Research Unit, Research and Innovation Centre, Fondazione Edmund Mach, San Michele All'Adige, Trento, Italy; b Applied Ecology Research Unit, Research and Innovation Centre, Fondazione Edmund Mach, San Michele All'Adige, Trento, Italy; c NBFC, National Biodiversity Future Center, Palermo 90133, Italy

**Keywords:** Zika virus, Re-emerging disease, Phylogeny, Virus phylodynamics, Molecular clock

## Abstract

**Background:**

Zika virus (ZIKV), a Flaviviridae family member, has been linked to severe neurological disorders. Despite detailed studies on recent outbreaks, the early evolutionary history of ZIKV remains partially unclear. This study elucidates ZIKV origin and evolutionary dynamics, focusing on recombination events, early lineage diversification, and virus spread across continents.

**Methods:**

We assessed recombination using multiple methods. We conducted Bayesian phylogenetic analyses to understand the evolutionary relationships and timing of key diversification events. Model selection was carried out to determine the most appropriate evolutionary model for our dataset.

**Results:**

Our phylogenies revealed recent recombination between Singaporean and African lineages, indicating the co-circulation of diverse lineages during outbreaks. Thailand was identified as a crucial hub in the spread across Asia. The phylogenetic analysis suggests that the ZIKV lineage dates back to the eleventh century, with the first significant diversification occurring in the nineteenth century. The timing of the re-introduction of the Asian lineage into Africa and the delay between probable introduction and outbreak onset were also determined.

**Conclusions:**

This study provides novel insights into ZIKV's origin and early evolutionary dynamics, highlighting Thailand's role in the spread of the virus in Asia and recent recombination events between distant lineages. These findings emphasize the need for continuous surveillance and a better understanding of ZIKV biology to forecast and mitigate future outbreaks.

## Highlights

• Recombination between African and Asian lineage in Singapore outbreak.
• Cell passage history can be a source of bias in estimating dated phylogeny.
• The study found that actual ZIKV lineages have likely been circulating in Thailand since at least 1999.
• Revision of dated analysis shows more recent estimates on the ZIKV diversification than previous work.
• ZIKV and SPOV divergence dates back to the 8th century.

## 1 Background

Zika virus (ZIKV) is a positive-sense ssRNA mosquito-borne Arbovirus belonging to the Flaviviridae family and flavivirus genus. ZIKV was first reported during a Yellow fever survey in Uganda in 1947 (Kirya, 1977; Kirya and Okia, 1977; Dick et al., 1952), while the first human case was reported in 1954 in Nigeria (Macnamara, 1954). Since its discovery and proven pathogenicity, this virus has been considered a neglected tropical disease involved in a few local outbreaks, mainly in the Pacific Islands, such as Yap Island in 2007 (Duffy et al., 2009), French Polynesia 2013 (Cao-Lormeau et al., 2014) and New Caledonia (Lanciotti et al., 2008; Ioos et al., 2014; Hayes, 2009). Other flavivirus species, such as Dengue, Yellow fever, and West Nile viruses, also pose significant global health threats, with similar mosquito-borne transmission patterns and increasing geographic spread due to climate change and urbanization (Gould and Solomon, 2008). ZIKV infection usually causes mild effects that last less than seven days. However, it can cause microcephaly and birth defects in the fetuses of infected pregnant women (Mlakar et al., 2016; Rasmussen et al., 2016; Ventura et al., 2016). In March 2015, ZIKV was first detected in Brazil (Zanluca et al., 2015), from which it spread all over the Americas (Zhang et al., 2017; Massad et al., 2017; Faria et al., 2016, 2017). The circulation history of ZIKV in Thailand has been long in recent years (Khongwichit et al., 2023; Phumee et al., 2023).

Several studies have addressed the dynamics of ZIKV evolution and circulation since the onset in 2015 in Brazil (Metsky et al., 2017; Faria et al., 2016; Musso, 2015) and even before, describing the diffusion of ZIKV before the main outbreaks in Yap Island and in Brazil (Gubler et al., 2017; Faye et al., 2014). Other works have described the timing of subsequent multiple introductions in Florida, Mexico, and the Caribbean islands (Grubaugh et al., 2017; Costa et al., 2020). Much information has been gathered in the last few years regarding its recent dynamics (Faria et al., 2017, 2016; Hu et al., 2019; Wang et al., 2016). Recent studies have suggested long-term circulation of this virus in Thailand (Ruchusatsawat et al., 2019; Phumee et al., 2023, 2019), India (Yadav et al., 2019), in China (Zhou et al., 2020) and other countries in Southeast Asia (Liu et al., 2019); ZIKV seroprevalence has been observed across Southeast Asia (Musso, 2015), where small epidemic events occurred between 2016 and 2019 (Bhargavi and Moa, 2020).

In addition, ZIKV infections linked to American strains have been reported in several African countries, such as Angola in 2016 (Hill et al., 2019) and Cape Verde between 2015 and 2016 (Faye et al., 2020). This highlights the potential for ZIKV to spread across the region. In recent years, Southeast Asia, particularly Thailand, and China have also seen a resurgence of ZIKV epidemics driven by local strains (Bahoussi et al., 2024; Khongwichit et al., 2023; Phumee et al., 2023; Zhou et al., 2020). Furthermore, a hidden circulation of ZIKV in Cuba was detected through surveys of travelers (Grubaugh et al., 2019). The re-emergence of ZIKV in Africa raises concerns, particularly due to the potential increased pathogenicity it may have developed (Liu et al., 2019; Pettersson et al., 2018; Aubry et al., 2021). Additionally, the American ZIKV strain has the potential to interact with various mosquito species in Africa, which may introduce new vectors into the infection transmission routes (Kauffman and Kramer, 2017; Weger-Lucarelli et al., 2016; Epelboin et al., 2017).

This article presents comprehensive results that shed light on the role of Southeast Asia, particularly Thailand, as an infection reservoir for the three outbreaks that have occurred thus far in Asia (Singapore, Yap Islands, and French Polynesia). The paper investigates the origin of ZIKV and the African–Asian split in detail. Other authors have already extensively investigated the ZIKV phylogeny on a shallow time scale and on a local scale, with particular attention given to its emergence in Brazil. Here, the authors want to address the need and the usefulness of a more comprehensive look into ZIKV evolution, investigating the origin of ZIKV and the pre-pandemic dynamics on a global scale and dating nodes that were not explored yet..

Studying flavivirus evolution is essential for both scientific understanding and public health purposes. The identification of flavivirus genome regions has the potential to serve as molecular targets for developing new strategies for contrasting Flaviviruses infection (Arenas, 2020). This can be achieved by analyzing phylodynamics to determine when and where specific mutations occurred. In ZIKV several amino acid substitutions have been linked to increased transmissibility to mosquito vectors (Liu et al., 2017; Shan et al., 2020) as well as enhanced, which is associated with fetal microcephaly (Yuan et al., 2017). Studing the evolution of the Flaviviridae family is essential for addressing emerging and re-emerging viral threats.

## 2 Methods

### 2.1 Dataset preparation

ZIKV sequences were downloaded from GenBank in June 2021, with a total of 1733 hits (Sayers et al., 2019). Sequences were filtered by the following criteria: GenBank files, including collection date, sampling location and sequences longer than 700 bp, were considered. Duplicates were filtered out, obtaining a dataset of 479 sequences.. By applying the same criteria, we downloaded five Spondweni viruses (SPOVs), only one of which satisfied the criteria (MG182017). Flanking regions (5′ and 3′ UTRs) were removed from the alignment due to the high variability and high amount of missing data. The second dataset was created by refining the previous one. First, we eliminated shorter sequences, keeping only those longer than 9000 nt. Next, we used a phylogenetic tree derived from the 479 sequences to further prune our dataset, reducing the overrepresented clades. This last step was done to reduce the node-density effect (Bromham et al., 2018; Hugall and Lee, 2007). and obtain a more suitable dataset to investigate the deeper phylogeny of ZIKV, narrowing the previous dataset to 117 sequences. The third dataset was obtained by adding the only SPOV sequence to estimate the origin of the ZIKV, with a total of 118 taxa. All the datasets were aligned using MAFFT (Katoh et al., 2019) (the alignments are provided by link).

### 2.2 Recombination

The dataset was analyzed with RDP v4.4.8 (Martin et al., 2015) to prevent possible phylogenetic biases due to recombination events. This software allows the analysis of the same datasets with different tools at the same time and includes different methods, such as GENECONV (Padidam et al., 1999), Chimera (Posada and Crandall, 2002), MaxChi, Bootscan (Salminen et al., 1995) and 3Seq (Boni et al., 2007). A recombination event was considered significant if it was detected by at least four out of five methods, with a p value ≤ 0.01, and the Bonferroni correction was applied to avoid false positives. IQ-TREE 1.6.12 (Nguyen et al., 2015) was used to assess the differences in evolutionary history between the recombinant sequence and the rest of the genome (for the phylogenetic methods, see Section 2.3, Methods). Recombinant viral sequences can influence molecular clock analyses and phylogenetic relationships because no single phylogenetic tree fully represents the genealogy of the sampled sequences (Schierup and Hein, 2000a, Schierup and Hein, 2000b; Bertrand et al., 2012). To avoid potential systematic bias in our phylogenetic analysis, we removed any sequences that show recombination patterns from the alignment (Duchêne et al., 2014, Rieux and Balloux, 2016).

### 2.3 Phylogenetic analysis

IQ-TREE 1.6.12 (Nguyen et al., 2015) was used for Maximum-Likelihood (ML) analysis. The ML phylogenies were obtained for all the datasets tested using ultrafast bootstrapping (Minh et al., 2013) with 1000 bootstrap alignments, 1000 maximum iterations, the approximate Bayes test and the SH-like approximate likelihood ratio test.

BEAST v2.6 was used to explore the timing of ZIKV evolution from the early divergence time to its origin (Bouckaert et al., 2014; Baele et al., 2013). We generated beast files with multiple clock models and tree priors combinations to assess which prior model performed better on the dataset. Two runs per combination were performed to check convergence. In total, we use seven prior combinations, relaxed clocks combined with three coalescent tree priors Coalescent Constant, Coalescent Exponential and Coalescent Bayesian Skyline. The same tree priors were used in combination with the strict clock. In addition, we employed the Birth-Death Serial (BDS) tree prior in combination with the relaxed clock to compare an outsider model to models that have more biological sense. All the clock priors were set with a minimum of 10^–5^ and a maximum of 10^–2^ using a uniform distribution. This value range is observed by Duffy et al. (2008) for RNA viruses, and it comprises the clock rate observed in a previous ZIKV study (Metsky et al., 2017; Pettersson et al., 2018; Duffy et al., 2008). The substitution model employed in every run was GTR + γ. All the chains were run for 200,000,000 generations until they reached convergence, which was assessed using Tracer 1.7 (Rambaut et al., 2018). All BEAST runs were calibrated with tip dates, where the most recent sample was set as zero time in the tree 15/10/2018. Moreover, we used the collection location as a discrete trait to infer the node's state in the phylogeny. To investigate ZIKV evolutionary dynamics, we employed three datasets. BEAST analysis was ran on each dataset employing the same priors and parameters suggested by the model selection. The reduced dataset containing 118 ZIKV sequences uses the collection location as a discrete trait to infer the node's ancestral state (Bouckaert, 2012).

### 2.4 Model selection

BEAST2 analyses were run using different sets of priors. Stepping Stone (SS) method was employed to compare all the evolutionary models applied in this study. SS allows for the comparison of different analyses using the marginal likelihood and the Bayesian factor. This type of model has several advantages over other alternatives, such as AICm and the harmonic mean (Baele et al., 2013). The MODEL SELECTION package was used to perform log marginal likelihood estimates for a different combination of the molecular clock and coalescent tree. Model selection was performed on every xml included in the analysis described above. The evolutionary models were tested along with both strict and uncorrelated relaxed clocks. In addition, we ran the model selection on the BDS to compare a speciation model against the coalescent models, which are supposed to be the most fitting models for describing ZIKV evolutionary dynamics. The Bayesian factor was calculated as described in the BEAST tutorial (Barido-Sottani et al., 2017).

## 3 Results and discussion

### 3.1 Datasets

A set of 479 sequences was obtained, which we used to construct three different datasets: (i) A larger dataset, the first employed in the analysis, containing 479 sequences of 10,811 nt lengths, with 28 sequences collected in Africa, 123 from Asia, 24 from the Pacific Islands and 305 from the Americas. Moreover, we carefully included in our datasets the French Polynesian sequence KX447518, which was found to be most closely related to the American outbreak by Pettersson (Pettersson et al., 2016, 2018), to obtain a comparable node for the origin of the American outbreak. (ii) The second dataset employed is a subsample of the first dataset, which includes 117 sequences in total, of which 8 African samples, 35 Asian samples, and 74 American samples. (iii) The dataset analyzed included 118 sequences. It was obtained by adding the only suitable SPOV sequence to the second dataset described here.

### 3.2 Signal of recombination detected between African and Asian lineages

**Fig. 1:**
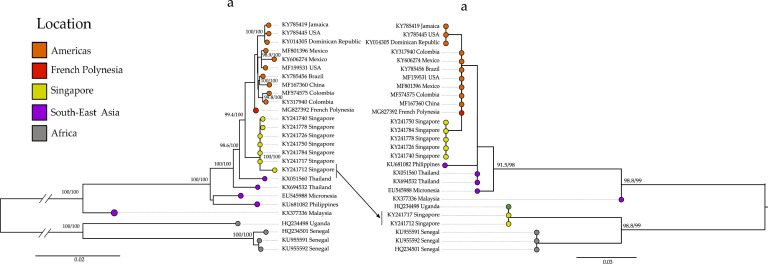
Phylogeny of recombinant and nonrecombinant regions in a Singapore sample. a) ML tree of the nonrecombinant region using IQ-tree using a subset of sequences: all the Singaporean sequences cluster together. b) ML tree of the recombinant region. The Singapore samples (KY241712 and KY241717) clustered with the African sequence. The tips are colored in accordance with the sampled location.

RDP4 analysis detected two recombination events, suggesting that the Singapore strain was a major contributor and that the Uganda strains (1947; Accession: HQ234498, 1962; Accession: KY288905) were minor contributors. A breakpoint was detected in the E genomic region, while another similar breakpoint was detected in the African sequences (Faye et al., 2014). RDP4 detected this recombination event with all the methods employed, given a p-value threshold of 0.01. The phylogenetic analysis highlighted the recombination event (see Fig. 1). The KY241717 and KY241717 samples collected in Singapore appear in different positions in the two trees, one using the recombinant region and one using the rest of the genome. According to the phylogenetic analysis of the recombinant region, the two Singapore samples clustered with the African clade with strong support (bootstrap support: 99; SH-aLRT: 98.8). The phylogenetic tree obtained from the remaining genome sequences placed the two recombinant samples with the remaining Singaporean samples. These sequences were excluded from further analysis to avoid systematic error (Posada and Crandall, 2002). This recombination event suggests co-circulation of an undetected African strain in Singapore, with secondary reintroduction of ZIKV in Asia. The recombination event in Flavivirus seems not to be as common as that in other groups of positive-stranded ssRNA(+) viruses (Taucher et al., 2010; Patiño-Galindo et al., 2021); however, recombination occurrence was observed especially in the Dengue virus group (Holmes et al., 1999; Simon-Loriere and Holmes, 2011; Tolou et al., 2001; Pérez-Losada et al., 2015) but seemed lower in ZIKV (Patiño-Galindo et al., 2021).

### 3.3 Most fitting clock priors from model selection

Marginal likelihood provided support for the relaxed molecular clock, with the best-fitting tree prior being a nonparametric Bayesian Skyline. Table 1 shows six combinations of parameters, which are ranked via the Bayes factor (BF) (Kass and Raftery, 1995). Following Kass and Raftery, the evidence strength of a hypothesis against H_0_ was rated as BF<3 = no evidence; 3–20 = positive support; 20–150 = strong support; and > 150 = overwhelming support (Kass and Raftery, 1995).

**Table 1:** The table shows the model selection using the Stepping Stone (SS) analysis. The marginal likelihood values were compared using the Bayes factor (BF), and the values were ranked in a readable format. The last column ranks the models based on the marginal likelihood analysis.

Clock prior	Tree Prior	Marginal Likelihood	BF*	BF^1^	Rank
Relaxed Clock	Coalescent Bayesian Skyline	−37,482.14905	108.05095	15.95496897	1
	Coalescent Constant	−37,489.91496	100.2850412	8.189060159	2
	Coalescent Exponential	−37,534.1891	56.01090227	−36.08507881	4
Strict clock	Coalescent Bayesian Skyline	−37,498.10402	92.09598107	–	3
	Coalescent Constant	−37,555.80642	34.39358112	−57.70239995	5
	Coalescent Exponential	−37,561.11131	29.08869762	−63.00728345	6
Relaxed Clock	Birth and Death Serial	−37,590.2321	–	−92.12808	7

⁎ Bayesian factor calculated using the Birth and Death Serial (BDS) as a comparison for other models.

1 Bayesian factor calculated using the Calescent Bayesian Skyline strict clock as a comparison for other models.

In Table 1, the lognormal uncorrelated clock using a coalescent tree prior is favored over the strict clock. The analysis slightly rejected the coalescent constant model (see Table 1), but the model provided similar tree topology and posterior estimates, as shown in Table 2 and Supplementary Fig. 1.

**Table 2:** Here, we report key results for the different models compared in the analysis: the mean clock rate, the root age and the American crown age are shown for all the combinations. For the model that accounts for the relaxed clock, the coefficient of variation (σ_r_)_,_ this parameter measures the clock likeliness of the rate. The Birth-Death Serial (BDS) model was chosen to represent the outliers. Coalescent models are preferred over speciation models such as BDS.

Clock prior	Tree Prior	Rank	Clock rate*	Root age (95 % HPD)	American crown (95 % HPD)	σ_r_
Relaxed Clock	CBS	1	7.01	135.2 (85.67 - 197.24)	5.96 (5.3 - 6.86) 2012/10/17	0.539
	CC	2	7.25	146.7 (86.07 - 227–62)	6.23 (5.49 - 7.2) 2012/07/16	0.624
	CE	4	7.22	107.8 (62.93 - 177.07)	6.25 (6.48 - 7.17) 2012/07/22	0.649
Strict clock	CBS	3	5.59	176.4 (159.8 - 193–03)	6.33 (5.9 - 6.79) 2012/06/19	–
	CC	5	5.63	178.5 (157.64 - 200.49)	6.48 (5.97 - 7.06) 2012/05/05	–
	CE	6	5.61	177.4 (158.48 - 199.69)	6.48 (5.98 - 7.06) 2012/05/05	–
Relaxed Clock	BDS	7	9.87	66.3 (55.42 - 79.84)	6.04 (5.33 - 6.92) 2012/9/10	0.783

⁎ mutation/site/year *10^–4^

Even though the Bayesian skyline tree prior is positively favored over the coalescent constant, the posterior estimates are consistent with each other. Moreover, we observed that models providing older node estimates are rejected along with the models that provide younger node estimates, showing that the data are better explained by a model that places the mean age of the tree at the end of the 19th century (Supplementary Fig. 1). The molecular clock does not vary much across the models employed in this study (Table 2), even though the selected clock (relaxed clock) shows a higher overall molecular rate than the strict clock.

In addition, the coefficient of variation (σ_r_; the standard deviation divided by the mean in Table 2) suggests that the relaxed clock assumption is theoretically sound since this parameter is estimated to be 0.53. σ_r_ measures the clock likeliness of the data; if σ_r_ is close to zero (lower than 0.2), then the data have low rate variation and can be modeled as a strict clock; vice versa, values that range between 0.2 and 1 show the relaxed clock assumption over the strict clock (Barido-Sottani et al., 2017; Drummond et al., 2006). The selected model was then employed in the analysis with SPOV as well.

### 3.4 Sample history affects the mutation rate

The phylogenetic analysis provides results for nodes that have not yet been studied. Investigating the deep nodes of ZIKV is challenging and requires specific care during data curation. In particular, several old samples from Africa were retained in the dataset in many studies, which can represent a possible source of error for the calibration analysis and become an issue for estimating the topology of the basal node, which is essential for establishing ZIKV's origin. Therefore, all samples showing a long or unspecified passage cell history were removed from the final dataset (Supplementary Table 2). An analysis was performed using a complete dataset to check the effect of cell passage history on the calibration and rates using BEAST2 (Supplementary Tree 1). The dataset included 479 sequences from old samples collected throughout the cell passage history. The rate behaves erratically in the tree obtained using a complete dataset with no filtering by cell passage or sample history.

Moreover, in the African lineage, the rate varies extensively across branches, and the log file shows that many parameters do not converge (ESS << 200). The σ^r^ measure is above one, which means that the temporal signal embedded in our alignment is highly random and does not follow any clock-like pattern. In such a scenario, the temporal signal is inconsistent for two main reasons: (i) evolution did not stop at the time of the reported collection date. Hence, employing the tip dating approach to these sequences can bias the posterior estimates; (ii) cell culture does not apply the same evolutionary pressure on viruses as the natural environment.

The selective pressure in some sites is relaxed and is no longer under purifying selection; hence, the passage history can affect the analysis (Haddow et al., 2012; Bush et al., 2000). In addition, the natural transmission bottleneck during Flavivirus infections decreases overall virus diversity, which does not occur in cell culture. Diversity reduction through transmission bottlenecks is well documented for mosquito-borne Flaviviruses, as is the effect of purifying selection by the host species on the virus (Forrester et al., 2014; Lequime et al., 2016; Grubaugh et al., 2016; Ciota et al., 2012; Fitzmeyer et al., 2023; Weaver et al., 2021). The transmission bottlenecks are missing in cell culture, providing an unrealistically high diversity. Eventually, cell culture passages promote artificial enhancement of the mutation rate, which is deleterious for evolutionary analysis. For these reasons, we decided to remove those sequences from the analysis.

### 3.5 Zika phylogeny

**Fig. 2:**
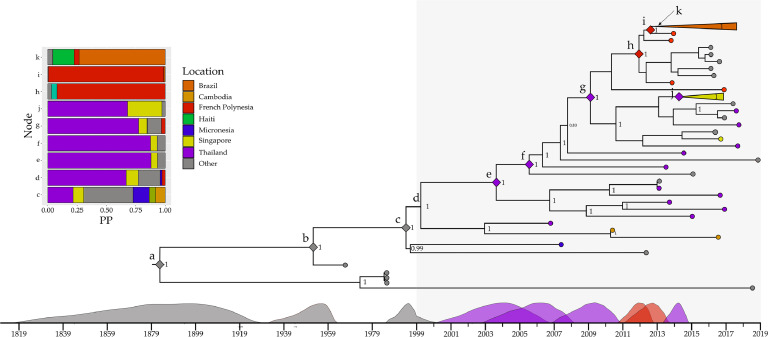
ZIKV phylogeny of a subset of 117 sequences. The figure has two different scales: the light grey box defines a magnified view of the recent phylogeny, and the dark grey bar represents the interval of node I determined by Metsky et al. (2017). The tips are coloured according to their corresponding collection locations in the legend. On the *x*-axis, the 95 % HPD is plotted; the color corresponds to the color of the node above. The histogram provides the posterior probability of the node location. The grey shading indicates a change in scale over time plotted on the *x*-axis.

Previous studies have shown that ZIKV diverged into two lineages at the beginning of its diversification: American and African lineages (Pettersson et al., 2018; Gong et al., 2017). The phylogenetic tree presented in Fig. 2 (paragraph 3.6) provides evidence for the monophyly of the African clade and the Asian clade. In addition, our African sampling can provide only a narrow view of the limited diversity of the African clade due to the unbalanced sampling, limiting our knowledge of the broad scenario of ZIKV evolution in Africa. Hence, more ZIKV sequences from the African continent are needed to better address this issue and try to answer Gong's question, ‘Zika virus two or three lineages?’ (Gong et al., 2017). Faye et al. (2014) contributed to this topic with a deep analysis of Africa's ZIKV circulation, although this question remains open (Faye et al., 2014). Moreover, African strains exhibit greater transmissibility and pathogenicity than strains of the Asian and American lineages (Aubry et al., 2021). Indeed, the low seroprevalence of ZIKV in Africa is explained by vector susceptibility and not by ZIKV transmissibility itself (Aubry et al., 2020).

### 3.6 Node age and trait estimates

According to the model selection, the Bayesian skyline coalescent tree prior is the best fitting model describing our data. The tree presented in Fig. 2 displays the phylogeny of ZIKV with the American and Singaporean outbreaks in a collapsed format. In addition, a histogram displaying the posterior probability (PP) is included for ancestral reconstruction analysis. Table 3 provides the posterior estimates for the node age, the 95 % HPD and the comparison with Patterson's estimates (Pettersson et al., 2018, 2016)

**Table 3:** Estimated node age for key events in ZIKV evolution. We provide the time expressed in years before the 0 time of the tree and in the date format (yyyy/mm), and the estimations are compared with the data provided by Patterson et al. (2018). Different taxon sampling methods do not allow for comparisons.

Node*	Age^1^	Age (yy/mm)	Age 95 % HPD	Age 95 HPD (yy/mm)	Petterson et al. 2018 (yy/mm)
a	135.2	1883/08	85.67 - 197.24	1933/03 - 1821/08	1834/8 (1852/08 – 1814/11)
b	66.4	1952/06	56.34 - 85	1962/07 - 1933/11	1948/03 (1953/10 – 1942/04)
c	24.8	1994/02	18.34- 33.98	2000/07 - 1984/11	1994/03 (1996/11 – 1991/08)
d	19.4	1999/06	15.14 −24.66	2003/09 - 1994/03	1999/02 (2001/04 – 1997/12)
e	15.03	2003/10	11.9 - 18.66	2006/10 - 2000/03	2003/10 (2005/06 – 2001/11)
f	13.12	2005/9	10.15 - 16.2	2008/09 - 2002/08	/
g	9.59	2009/4	7.58 −11.71	2011/04 - 2007/02	/
h	8.36	2012/2	5.87 - 7.99	2012/10 - 2010/11	2012/04 (2012/17 – 2011/08)
i	6.18	2012/9	5.46 - 7.12	2013/05 - 2011/09	2012/10 (2013/02 – 2012/03)
k	5.97	2012/10	5.3–6.81	2013/07 - 2012/02	2012/11 (2013/04 – 2012/05)^2^
j	4.56	2013/11	3.8 - 5–4	2015/02 - 2013/06	/
Angola radiation	3.76	2015/2	2.58 - 4.72	2016/04 - 2014/03	/
Cape - America split	4.6	2014/4	4.14 - 5.19	2014/08 - 2013/08	/
Cape radiation	3.65	2015/3	3.19 - 4.27	2015/08 - 2014/08	/

⁎ (pp>0.95).

1 Age is provided in years before the most recent collected sample 15/10/2018.

2 This node defines the American ZIKV radiation, but the topology provided by Pettersson and by us is different due to low node support within the American outbreak.

The African and Asian-American clades (node *a*) diverged approximately 135 years before the most recent sample, in other words, in 1883 (HPD 95 % 1933/03 - 1821/08). The African and Asian lineage estimates partially overlap with the estimate in Pettersson, 1834–08 (1814–11 – 1852–08) (see Table 3). Pettersson's estimates employed a strict clock prior; this can explain the differences between our findings and Patterson's estimates (Pettersson et al., 2018). Node *b* represents the Asian radiation that occurred in 1952 (HPD 95 % 1962/07 - 1933/11), indicating that ZIKV was already circulating in Southeast Asia at that time.

This estimate supports previous findings about the emergence of ZIKV in Asia between the forties and fifties (Pettersson et al., 2016, 2018; Faria et al., 2016). The ancestral node reconstruction for nodes *a* and *b* did not provide a clear result because of the signal paucity contributing to the inference of the ancestral state. Node *c* (1994/02 HPD 95 % 2000/07 - 1984/11) shows the coalescent event of all the lineages involved in recent epidemics (Yap Island, Singapore and the Americas). The role of these lineages in local outbreaks makes sense in light of Pettersson's findings, in which a mutation in the E protein is detected. This particular mutation has been shown to be closely associated with the enhanced spreading potential of Flaviviruses (Fritz et al., 2011). The histogram in Fig. 2 provides the ancestral state reconstruction for this node. However, the posterior probability (PP) does not favor one location over another.

In the upper nodes, the analysis revealed that Thailand played a pivotal role in the source of the epidemic in Asia (see the histogram in Fig. 2). Node *d* has a posterior probability (PP) of 0.67 of being of Thailand origin, which is estimated to be in 1999/06 (HPD 95 % 2003/09 - 1994/03). Ancestral inference places nodes *d, e, f, g* and *j* in Thailand with high PP (d: 0.67; e: 0.88; f: 0.88; g: 0.77; j: 0.68). These data are supported by observational studies that address the long circulation of ZIKV in Thailand (Buathong et al., 2015; Ruchusatsawat et al., 2019; Khongwichit et al., 2023). The earliest sequence in Thailand was collected in 2006/10/28 (MG645981), and it is the second oldest sequence of ZIKV in Asia after the Malaysian sample dated 1966. These findings are indicative of the presence of ZIKV in Southeast Asia long before its first detection, suggesting that long-term ZIKV adaptation to the local environment occurred between the second half of the 20th century and 2006 (Noisumdaeng et al., 2023; Pond, 1963).

According to the phylogenetic analysis, in recent years, many Southeast Asian countries, including Singapore, Indonesia, India, and Cambodia, have reported infections originating from Thailand. The evidence suggested that ZIKV likely circulated in the country 20 years ago, in 1999/06 (HPD 95 % 2003/09 - 1994/03), node *d.* Furthermore, other cases of introduction (Japan, Europe, China) were reported to be connected with tourism but never arose into local outbreaks in the country where the virus was imported.

Node *j* highlights the split between a Thailand sample and the Singapore lineage; the split is estimated to have occurred approximately 2014/4 (HPD 95 % 2015/02 - 2013/06), whereas the Singapore lineage started to diversify at 2014/8 (HPD 95 % 2015/5 – 2014/1). Although ZIKV seems to have started circulating in Singapore earlier than 2015/5, the infection cluster was detected only in August 2016 (Maurer-Stroh et al., 2016; Singapore Zika Study Group, 2017). In addition, two different lineages were detected during the Singapore outbreak: the main lineage (yellow triangle in Fig. 2) and another sample related to the Thailand sequences; this evidence reveals that two independent ZIKV introductions in Singapore contributed to the outbreak.

Patterson observed the same phenomenon between phylogeny employing the African outgroup or omitting it. The MRCA estimates for the *k* and *i* nodes provided here are 2012/10 (HPD 95 % 2013/07 - 2012/02) and 2012/9 (HPD 95 % 2015/02 - 2013/06), respectively. These posterior estimates presented here represent older estimates than other previously proposed dates suggested by Faria and Metsky, which date the introduction of ZIKV in the Americas between October 2013 and April 2014. The differences among estimates are probably due to differences in taxon sampling. Indeed, the analyses of Faria and Metsky focused on the American outbreak (Faria et al., 2017, 2016; Metsky et al., 2017). Our dataset can provide better results for analyzing events that led to American and Singaporean outbreaks since it is less skewed toward America's epidemic. Our dataset contains all the suitable sequences of ZIKV in East Asia, and we selected a subsample of American sequences. Our dataset contains samples that break the long branches between node *b* and node *i*. This approach increases the accuracy of branch-length estimates by reducing the node-density effect and the variance of the estimates (Bromham et al., 2018; Hugall and Lee, 2007).

Notably, outbreaks in Singapore and South America were reported long after the estimated introduction of ZIKV. This suggests long circulation of ZIKV before the onset of the outbreak, supporting an ecological scenario in which the virus takes time to start circulating consistently in a new population. The duration of an emerging epidemic is proportional to the basic reproductive number of the virus. Therefore, this delay between molecular estimation and timely detection of potential emerging outbreaks is extremely important for preventing further circulation of the virus, making prevention measures efficient before the infection is out of control and leading to an epidemic. Waiting until the number of infections is so high that the silent spread of an infectious disease becomes clear is too late. Prevention, vector control, and monitoring are the only ways to address future epidemics and manage emerging diseases. Constantly testing the mosquito vector for the presence of the target virus could help identify the potential onset of a new outbreak. For instance, Dengue virus (DENV) has been detected in vectors in Brazil, the Philippines and other countries where DENV is endemic (Figueiredo et al., 2010; Reis et al., 2019; Balingit et al., 2020; Lau et al., 2015).

### 3.7 The timing of the reintroduction of ZIKV in Africa

Reintroductions of ZIKV in Africa were detected in Angola and Cape Verde. In Angola, ZIKV was confirmed in 2016/12, but evidence from the previous circulation set a probable first case in 2016/9 (Hill et al., 2019; Neto et al., 2022). Our analysis points to the divergence of all Angolan sequences in 2015/2 (HPD 95 % 2016/4–2014/3) (see Table 3), indicating that ZIKV was circulating before this date. It is not possible to provide with precision the date of divergence of the Angolan samples from the most closely related American sample due to the low support at the nodes, but it appears to have occurred in mid-2014. The timing of the introduction of ZIKV to Cape Verde should fall within the time span between the American-Cape Verde split and Cape Verde radiation. These two events are estimated to have occurred in 2014/4 (HPD 95 % 2014/8–2013/08) and 2015/3 (HPC 95 % 2015/8–2014/8), whereas the first reported case of the epidemic occurred in October 2015 (Faye et al., 2020; Ward et al., 2022). The results provided here show that the Asian strain of ZIKV was reintroduced in Africa (Cape Verde and Angola) before the epidemic in Brazil was confirmed in May 2015. This suggests that the spread of ZIKV out of South America likely started before the ZIKV epidemic was detected in Brazil. These results emphasize the delay in detecting wide-reaching ZIKV circulation. The delay in detecting ZIKV onset has been a constant characteristic of all outbreaks in recent decades.

### 3.8 Origin of Zika and its divergence from Spondweni

The origin and divergence time of the deep nodes involved in ZIKV evolution have not yet been sufficiently investigated. This section tries to answer some still-open questions about how old the ZIKV is currently circulating. SPOV, the sister group of ZIKV, can infect humans, and its consequences are usually mild and less dangerous than those of ZIKV.

**Fig. 3:**
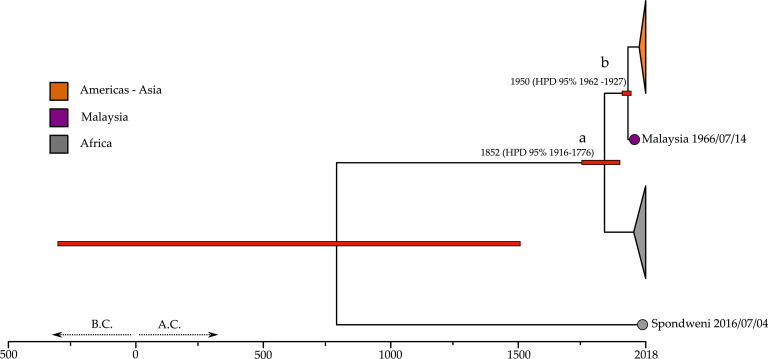
ZIKV phylogeny of a subset of 117 sequences and an additional SPOVi virus sequence as an outgroup. Here, we used the same prior set of the best fitting models, as shown in Table 1. The legend is consisten with the color of the two previous figures using only three colors to highlight the location of interest. Node *a* shows a wider range in the 95 % HPD than in the ZIKV phylogeny obtained with 117 ZIKV sequences, probably due to the effect of the outgroup.

Our results date the origin of ZIKV to ∼800 (HPD 95 % 1516 A.D. - 294 BC.), suggesting that the Middle Ages were the probable origin of ZIKV in Africa. The estimate for the Asian-African split of ZIKV (node *a*) is 1852 (HPD 95 % 1916–1776) (Fig. 3). The ZIKV diversification date estimates considerably overlap with the posterior data of our previous analysis, indicating that adding the SPOV outgroup does not bias the analysis. The only estimates for the age of the node in question are reported by Pettersson and Fiz-Palacios (2014); however, they are much older than those reported here. This is probably due to the wider taxon sampling and the calibration employed in the analysis that aimed to date the whole Flavivirus evolution, not only the ZIKV origin.

The clock rate estimated in the SPOV tree is 6.42 10^–4^ (m s^-1^/y), which is in line with the clock rate of Flavivirus and +ssRNA viruses, which are supposed to range between 10 and 3 and 10^–4^ (m s^-1^/y) (Duffy et al., 2008). Moreover, the clock estimated here is slower than the values calculated using only the outbreak sequences, approximately 10^–3^ (Fajardo et al., 2016; Faria et al., 2017; Metsky et al., 2017), as expected because the clock depends on the timespan of the phylogeny (Ho et al., 2011).

The divergence between ZIKV and SPOV occurred long before ZIKV diversification. The ZIKV lineage and the SPOV lineage probably underwent many evolutionary novelties in their lineage, and it cannot be excluded that other viruses related to these two lineages may still remain undetected but circulate via the mosquito vector infection route. This partially explains the high ZIKV seroprevalence in countries where ZIKV is endemic (Musso, 2015).

In conclusion, we want to stress a limitation in inferring the date of the ZIKV-SPOV split. The taxon sampling in this phylogenetic analysis is greatly biased toward ZIKV, and we only employed one SPOV sequence, which is the only one suitable for our analysis. This could affect the inference of the ZIKV-SPOV estimate by increasing the variance of the clock estimates. Indeed, the 95 % HPD of this node is wide at ∼800 (95 % HPD 294 BC. – 1516 A.D.). We cautiously suggest this timescale for ZIKV origin, as more sequences could increase the node density throughout the branches, leading to the SPOV and ZIKV lineages.

## 4 Concluding remarks

Interest in ZIKV has come and gone, leaving behind unanswered questions that we partially tried to address here. Our phylogenies provide useful information about the origin and early diversification of ZIKV. The most important topic discussed in this work is the evident delay in detecting ZIKV before outbreak onset: ZIKV circulated within communities for at least one year before outbreaks became evident in Brazil, Angola and Cape Verde. This unmanaged viral spread has led to sequential outbreaks (e.g., the spread of ZIKV in Micronesia, French Polynesia, the Americas, Angola and Cape Verde). The ancestral state reconstruction analysis clarified the role of Thailand in sustaining ZIKV circulation in Southeast Asia and its sequential outbreaks in Asia and the Americas.

ZIKV evolution must be paired with its vector spread and distribution. ZIKV is transmitted by *Aedes aegypti* and, less efficiently, by *Aedes albopictus;* these two major vectors are spreading all over the globe, bearing their infection potential (Kraemer et al., 2019, 2015). Climate change and rising temperatures in temperate regions can favor emerging diseases. The efficiency of virus transmission for these two species has been shown to correlate with environmental temperature (Reinhold et al., 2018; Chouin-Carneiro et al., 2020). Circulation of ZIKV can lead to further outbreaks if not controlled and monitored constantly, even in areas where it is not yet endemic. In recent years, Europe has experience several local infections with DENV and Chikungunya viruses (Lazzarini et al., 2020; Laverdeur et al., 2024; Monge et al., 2020; Grandadam et al., 2011; Caputo et al., 2020). These events were possibly due to the presence of vectors, increased connectivity and globalization, and they may have been favored by climate change. The DENV distribution is, for example, correlated not only with vector presence but also with climate factors such as rainfall, temperature and humidity (Laverdeur et al., 2024; Brady et al., 2014; Schaffner and Mathis 2014; Giunti et al., 2023). Current evidence suggests that climate change has partially guided recent outbreaks of several arboviruses (Medlock et al., 2015; Brugueras et al., 2020; Iwamura et al., 2020; Gould and Higgs, 2009). Reducing the spread of invasive vectors worldwide is essential for preventing new viral threats. Our results on the early evolution of the ZIKV reinforce the idea that increasing anthropic impact and natural niche disruption due to human activities, together with a globalized society favouring the mobility of people among different countries, are favouring the emergence of novel arbovirus threats.

## Funding

Not applicable.

## CRediT authorship contribution statement

**Nicola Zadra:** Writing – review & editing, Writing – original draft, Methodology, Investigation, Formal analysis, Data curation, Conceptualization. **Annapaola Rizzoli:** Writing – review & editing, Funding acquisition, Conceptualization.

## Declaration of competing interest

The authors declare that they have no known competing financial interests or personal relationships that could have appeared to influence the work reported in this paper.

## Data Availability

The datasets generated and/or analysed during the current study are available in the https://figshare.coms-1/3584edafdc1a4ff9ca4e repository.

## Appendix Supplementary materials

Image, application 1

Image, application 2

Image, application 3

Image, application 4
