# Supplementary material for: Comprehensive phylogenomic analysis of Zika virus: Insights into its origin, past evolutionary dynamics, and global spread
Source: Virus Res. 2024 Nov 8;350:199490. doi: 10.1016/j.virusres.2024.199490 (PMC11583807; doi:10.1016/j.virusres.2024.199490)
Supplement: Supplementary file 1 [file mmc1.pdf]

Supplementary Figure 1

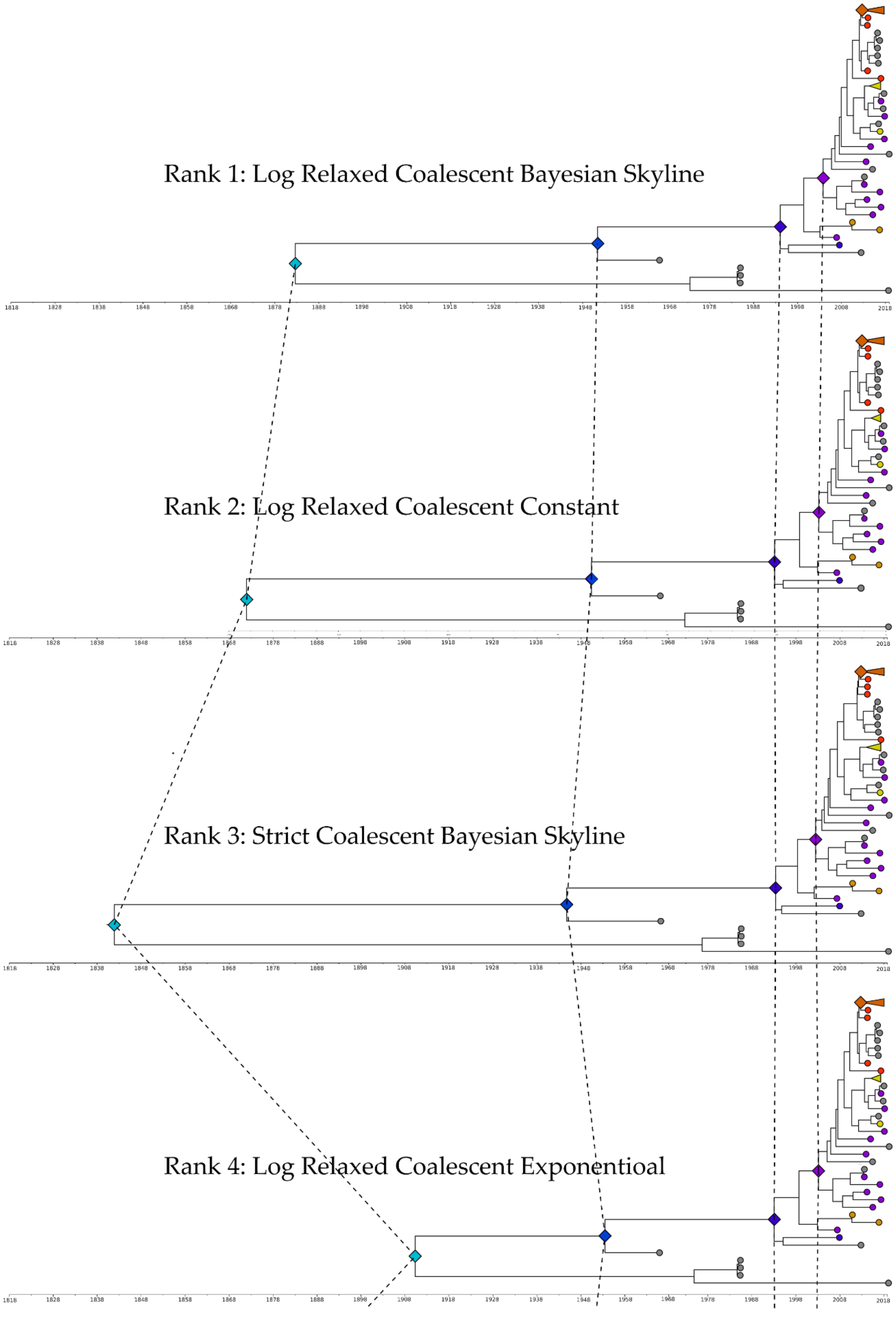

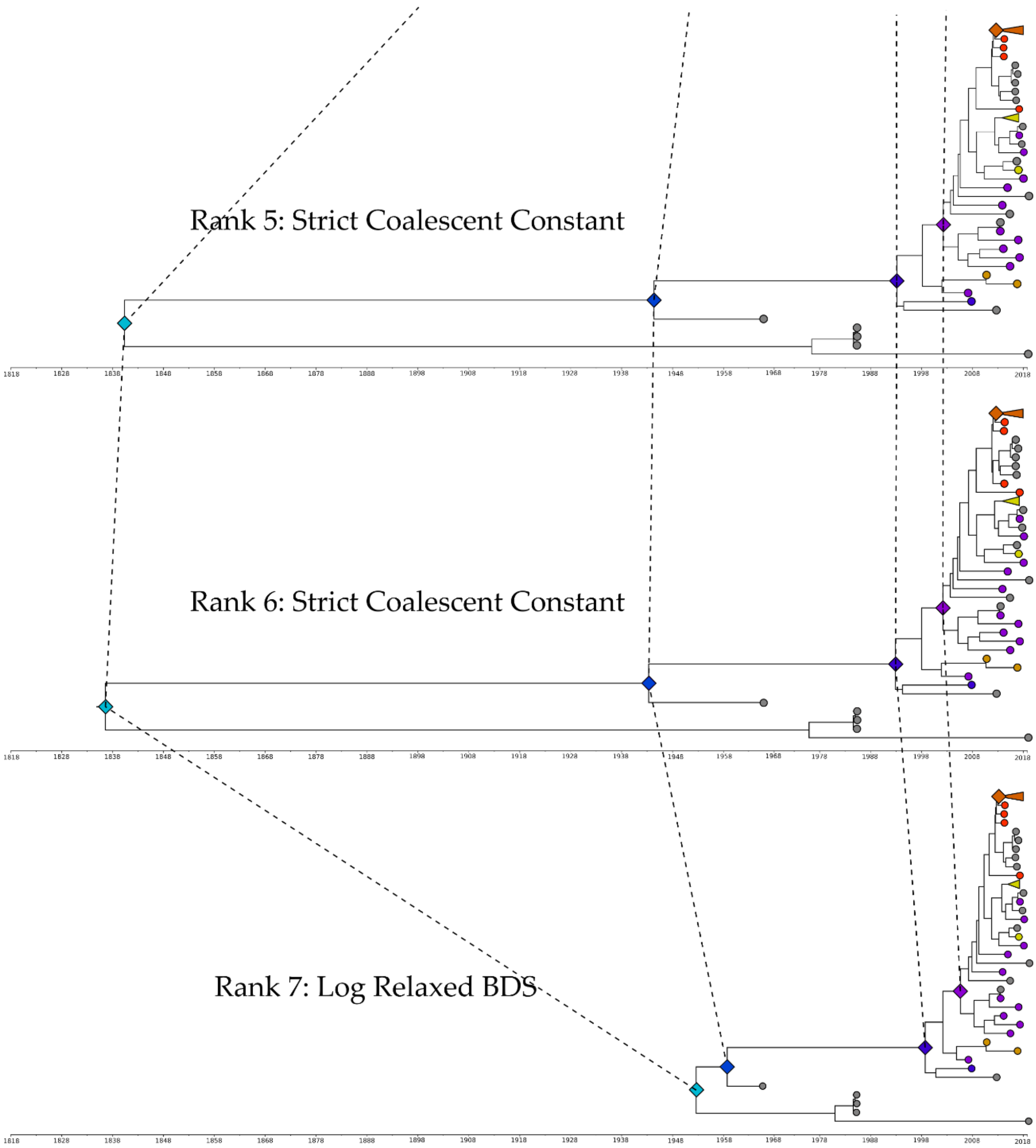

Supplementary Figure 1. Here we show graphically how the time tree changes across the different models, as in Table 1 the differences are more stressed in the deep nodes than in the upper. As detected in the model selection, the differences found between the tree rank 1 and the tree rank 2 are negligible; indeed, the trees look very similar in all the topology and in the posterior distribution of all the values.
