# Supplementary material for: Comprehensive phylogenomic analysis of Zika virus: Insights into its origin, past evolutionary dynamics, and global spread
Source: Virus Res. 2024 Nov 8;350:199490. doi: 10.1016/j.virusres.2024.199490 (PMC11583807; doi:10.1016/j.virusres.2024.199490)
Supplement: Supplementary file 2 [file mmc2.pdf]

Accession	Location	Collection Date
EU545988	Micronesia	15/6/2007
JN860885	Cambodia	1/6/2010
KF268948	Central African Republic	1/6/1976
KF270886	Gabon	1/6/2007
KF270887	Gabon	1/6/2007
KF383022	Senegal	1/6/1997
KF383029	Senegal	1/6/2002
KF383031	Senegal	1/6/1969
KF383034	Senegal	1/6/1979
KF383036	Cote d'Ivoire	1/6/1999
KF383037	Cote d'Ivoire	1/6/1996
KF383038	Cote d'Ivoire	1/6/1999
KF383039	Senegal	1/6/1991
KF383045	Cote d'Ivoire	1/6/1990
KF383046	Cote d'Ivoire	1/6/1999
KF383085	Senegal	1/6/1969
KF383086	Cote d'Ivoire	1/6/1999
KF383106	Cote d'Ivoire	1/6/1990
KF383114	Senegal	1/6/1979
KF993678	Canada	19/2/2013
KJ634273	Cook Islands	1/6/2014
KJ776791	French Polynesia	28/11/2013
KJ873160	New Caledonia	3/4/2014
KJ873161	New Caledonia	2/4/2014
KM078930	Chile Easter Island	4/4/2014
KM07897	Chile Easter Island	21/4/2014
KM851038	Philippines	9/5/2012
KR872956	Brazil	1/6/2015
KU179098	Indonesia Jambi	30/12/2014
KU312312	Suriname	2/10/2015
KU321639	Brazil	15/3/2015
KU365777	Brazil	1/6/2015
KU365778	Brazil	1/6/2015
KU365779	Brazil	1/6/2015
KU365780	Brazil	1/6/2015
KU497555	Brazil	30/11/2015
KU501215	Puerto Rico	1/12/2015
KU509998	Haiti	12/12/2014
KU527068	Brazil Rio Grande do Norte Natal	1/6/2015
KU647676	Martinique	15/12/2015
KU681081	Thailand	19/7/2014
KU681082	Philippines	9/5/2012
KU707826	Brazil	1/7/2015
KU729217	Brazil	1/6/2015
KU729218	Brazil	1/6/2015
KU744693	China	6/2/2016
KU758877	French Guiana	15/12/2015
KU761560	China	15/2/2016
KU761561	China	15/2/2016
KU761564	China	12/2/2016

KU820897	Colombia Barranquilla	15/12/2015
KU820899	China	17/2/2016
KU853013	Italy Padua	1/2/2016
KU870645	USA	2/2/2016
KU922923	Mexico Chiapas	25/2/2016
KU922960	Mexico Chiapas	25/2/2016
KU926309	Brazil	14/1/2016
KU926310	Brazil	29/1/2016
KU937936	Suriname	11/2/2016
KU940224	Brazil	1/8/2015
KU940228	Brazil	1/7/2015
KU955589	China	16/2/2016
KU955591	Senegal	20/11/1984
KU963574	Nigeria Ibadan	9/9/1968
KU963796	China Shenzhen	1/6/2016
KU991811	Italy Rome	6/3/2016
KU997667	China	16/2/2016
KX013000	China	17/2/2016
KX051560	Thailand	9/7/2013
KX051561	Thailand	21/9/2013
KX051562	Thailand	16/1/2015
KX051563	USA	5/2/2016
KX087101	Puerto Rico	15/12/2015
KX087102	Colombia Barranquilla	15/12/2015
KX117076	China	17/2/2016
KX156774	Panama	18/12/2015
KX156775	Panama	11/12/2015
KX156776	Panama	18/12/2015
KX197192	Brazil	1/6/2015
KX198135	Panama	1/6/2016
KX216632	Solomon Islands	1/6/2015
KX216634	Tonga	1/6/2016
KX216636	Samoa	1/6/2016
KX216639	Samoa	1/6/2016
KX216640	Cook Islands	1/6/2014
KX247632	Mexico	15/11/2015
KX247646	Colombia	9/2/2016
KX253996	China	16/2/2016
KX262887	Honduras	6/1/2016
KX266255	China	14/2/2016
KX280026	Brazil	1/6/2015
KX369547	French Polynesia	25/10/2013
KX377336	Malaysia	15/7/1966
KX380263	Fiji	1/6/2016
KX421194	Nicaragua	13/1/2016
KX421195	Nicaragua	19/1/2016
KX446950	Mexico Chiapas	1/1/2016
KX446951	Mexico Chiapas	1/1/2016
KX447509	French Polynesia	15/12/2013
KX447510	French Polynesia	15/12/2013
KX447511	French Polynesia	15/1/2014

KX447512	French Polynesia	15/12/2013
KX447513	French Polynesia	15/12/2013
KX447514	French Polynesia	15/1/2014
KX447515	French Polynesia	15/11/2013
KX447516	French Polynesia	15/1/2014
KX447517	French Polynesia	15/1/2014
KX447518	French Polynesia	15/12/2013
KX520666	Brazil Salvadore Bahia	15/8/2015
KX548902	Colombia	7/10/2015
KX601168	Puerto Rico	1/12/2015
KX601169	Uganda Entebbe	20/4/1947
KX673530	United Kingdom	21/4/2016
KX694532	Thailand	12/2/2013
KX694534	Honduras	6/1/2015
KX702400	Venezuela Barquisimeto	25/3/2016
KX766028	Dominican Republic	6/6/2016
KX766029	Mexico	23/6/2016
KX806557	Australia	15/2/2016
KX811222	Brazil Fortaleza	14/6/2016
KX813683	Singapore	27/8/2016
KX827268	USA	1/1/2016
KX827309	Singapore	28/8/2016
KX830930	Brazil	1/3/2016
KX832731	USA Miami Florida	24/8/2016
KX838904	USA Miami Florida	22/8/2016
KX838905	USA Miami Florida	23/8/2016
KX838906	USA Miami Florida	23/8/2016
KX842449	USA Miami Florida	22/6/2016
KX856011	Mexico Chiapas	1/1/2016
KX879603	Ecuador Esmeraldas	15/4/2016
KX879604	Ecuador Esmeraldas	15/4/2016
KX893855	Venezuela	25/3/2016
KX906952	Honduras	16/4/2016
KX922706	USA Miami Florida	5/8/2016
KX922707	USA Florida	17/8/2016
KY003152	Philippines Iloilo City	1/9/2016
KY003153	Italy	15/4/2016
KY003154	Italy	15/4/2016
KY014295	USA Florida	22/6/2016
KY014296	Brazil Rio de Janeiro	18/4/2016
KY014297	Brazil Rio de Janeiro	12/4/2016
KY014299	USA Miami Florida	4/9/2016
KY014300	Dominican Republic Distrito Nacional	20/4/2016
KY014302	Dominican Republic Distrito Nacional	21/4/2016
KY014303	Dominican Republic Distrito Nacional	11/4/2016
KY014304	Dominican Republic Distrito Nacional	18/4/2016
KY014305	Dominican Republic Distrito Nacional	5/4/2016
KY014306	Honduras Francisco Morazan	10/6/2016
KY014307	Brazil Rio de Janeiro	28/3/2016
KY014309	Brazil Rio de Janeiro	28/3/2016
KY014310	Honduras Francisco Morazan	9/5/2016

KY014312	Honduras Francisco Morazan	13/5/2016
KY014314	Dominican Republic Distrito Nacional	14/6/2016
KY014315	Honduras Francisco Morazan	6/6/2016
KY014316	USA Florida	17/8/2016
KY014317	Brazil Rio de Janeiro	21/3/2016
KY014318	Dominican Republic Distrito Nacional	27/4/2016
KY014319	Honduras Francisco Morazan	30/4/2016
KY014320	Brazil Rio de Janeiro	23/3/2016
KY014321	Dominican Republic Distrito Nacional	11/4/2016
KY014322	USA Miami Florida	23/8/2016
KY014323	USA Miami Florida	23/8/2016
KY014324	USA Miami Florida	22/8/2016
KY014325	USA Florida	2/8/2016
KY014327	Honduras Francisco Morazan	9/6/2016
KY075932	USA Florida	22/3/2016
KY075933	USA Florida	21/6/2016
KY075934	USA Florida	3/7/2016
KY075937	USA Florida	9/9/2016
KY075938	USA Florida	20/9/2016
KY075939	USA Florida	5/10/2016
KY120348	Mexico Oaxaca Tehuantepec Isthmus	3/3/2016
KY120349	Mexico Oaxaca Tehuantepec Isthmus	3/3/2016
KY126350	Taiwan	15/10/2016
KY126351	Taiwan	15/5/2016
KY131442	Vietnam Long An	15/6/2013
KY241671	Singapore	4/9/2016
KY241673	Singapore	4/9/2016
KY241674	Singapore	5/9/2016
KY241675	Singapore	6/9/2016
KY241676	Singapore	6/9/2016
KY241678	Singapore	7/9/2016
KY241679	Singapore	7/9/2016
KY241680	Singapore	7/9/2016
KY241681	Singapore	8/9/2016
KY241682	Singapore	8/9/2016
KY241683	Singapore	8/9/2016
KY241684	Singapore	8/9/2016
KY241685	Singapore	12/9/2016
KY241686	Singapore	12/9/2016
KY241687	Singapore	13/9/2016
KY241688	Singapore	13/9/2016
KY241689	Singapore	13/9/2016
KY241690	Singapore	13/9/2016
KY241691	Singapore	13/9/2016
KY241692	Singapore	14/9/2016
KY241694	Singapore	19/9/2016
KY241695	Singapore	20/9/2016
KY241696	Singapore	30/8/2016
KY241697	Singapore	27/8/2016
KY241698	Singapore	26/8/2016
KY241700	Singapore	27/8/2016

KY241704 Singapore	27/8/2016
KY241706 Singapore	28/8/2016
KY241707 Singapore	28/8/2016
KY241713 Singapore	28/8/2016
KY241714 Singapore	28/8/2016
KY241716 Singapore	28/8/2016
KY241719 Singapore	29/8/2016
KY241720 Singapore	29/8/2016
KY241721 Singapore	28/8/2016
KY241722 Singapore	28/8/2016
KY241725 Singapore	26/8/2016
KY241726 Singapore	28/8/2016
KY241727 Singapore	28/8/2016
KY241729 Singapore	29/8/2016
KY241730 Singapore	27/8/2016
KY241731 Singapore	30/8/2016
KY241733 Singapore	28/8/2016
KY241734 Singapore	28/8/2016
KY241736 Singapore	29/8/2016
KY241737 Singapore	28/8/2016
KY241738 Singapore	30/8/2016
KY241740 Singapore	29/8/2016
KY241741 Singapore	28/8/2016
KY241744 Singapore	28/8/2016
KY241748 Singapore	28/8/2016
KY241749 Singapore	28/8/2016
KY241750 Singapore	28/8/2016
KY241751 Singapore	28/8/2016
KY241753 Singapore	26/8/2016
KY241754 Singapore	7/9/2016
KY241756 Singapore	5/9/2016
KY241758 Singapore	30/8/2016
KY241763 Singapore	2/9/2016
KY241766 Singapore	15/9/2016
KY241767 Singapore	7/9/2016
KY241772 Singapore	30/8/2016
KY241773 Singapore	7/9/2016
KY241774 Singapore	5/9/2016
KY241775 Singapore	5/9/2016
KY241776 Singapore	5/9/2016
KY241777 Singapore	6/9/2016
KY241778 Singapore	6/9/2016
KY241779 Singapore	6/9/2016
KY241780 Singapore	6/9/2016
KY241781 Singapore	6/9/2016
KY241782 Singapore	7/9/2016
KY241783 Singapore	7/9/2016
KY241784 Singapore	8/9/2016
KY241785 Singapore	5/9/2016
KY241786 Singapore	6/9/2016
KY241787 Singapore	7/9/2016

KY241788 Singapore	13/5/2016
KY272987 Thailand	30/8/2016
KY272991 Brazil	12/2/2016
KY288905 Uganda	15/11/1962
KY293644 New Zeland	21/2/2016
KY317936 Colombia	16/1/2016
KY317937 Colombia	7/1/2016
KY317938 Colombia	10/1/2016
KY317940 Colombia	9/1/2016
KY325464 USA Florida	19/9/2016
KY325465 USA Florida	28/9/2016
KY325467 USA Florida	29/7/2016
KY325468 USA Florida	4/8/2016
KY325469 USA Florida	4/8/2016
KY325472 USA Florida	23/8/2016
KY325473 USA Florida	31/8/2016
KY325476 USA Florida	11/10/2016
KY325477 USA Florida	3/10/2016
KY325479 USA Florida	28/9/2016
KY328289 Honduras	15/5/2016
KY348640 Suriname	22/1/2016
KY354186 Brazil Manguinhos Rio de Janeiro	15/4/2015
KY379148 China	29/2/2016
KY415988 Haiti	5/6/2014
KY415990 Haiti	2/6/2014
KY415991 Haiti	24/6/2014
KY441401 Brazil	29/2/2016
KY441402 Brazil	5/4/2016
KY441403 Brazil	11/1/2016
KY553111 South Korea	15/4/2016
KY558999 Pernambuco	10/7/2016
KY559012 Brazil Ribeirao Preto Sao Paulo	19/4/2016
KY576904 Central African Republic	1/6/1989
KY606271 Mexico Chiapas	5/3/2016
KY606272 Mexico Oaxaca	5/8/2016
KY606273 Mexico Guerrero	30/6/2016
KY606274 Mexico Guerrero	7/7/2016
KY631492 Brazil Manaus Amazon State	8/1/2016
KY631493 Mexico Tapachula Chiapas	15/10/2015
KY631494 Mexico Tapachula Chiapas	15/10/2015
KY648934 Mexico Chiapas	1/6/2016
KY693676 Honduras	26/8/2016
KY693677 Honduras	26/8/2016
KY693678 Peru	28/6/2016
KY693679 Peru	11/7/2016
KY693680 Venezuela	19/10/2016
KY765317 Nicaragua Managua	29/6/2016
KY765318 Nicaragua Managua	10/7/2016
KY765320 Nicaragua Managua	29/5/2016
KY765321 Nicaragua Managua	10/7/2016
KY765322 Nicaragua Managua	29/6/2016

KY765323	Nicaragua Managua	30/6/2016
KY765324	Nicaragua Managua	7/7/2016
KY765326	Nicaragua Managua	30/6/2016
KY765327	Nicaragua Managua	22/4/2016
KY785415	Dominican Republic Distrito Nacional	11/4/2016
KY785418	Honduras Francisco Morazan	13/5/2016
KY785419	Jamaica	13/6/2016
KY785420	Dominican Republic Distrito Nacional	18/4/2016
KY785422	USA Miami Florida	9/9/2016
KY785423	Dominican Republic Distrito Nacional	5/4/2016
KY785427	Brazil Rio de Janeiro	30/3/2016
KY785429	Brazil Rio de Janeiro	14/4/2016
KY785435	Dominican Republic	7/7/2016
KY785441	Dominican Republic Distrito Nacional	13/6/2016
KY785442	Honduras Francisco Morazan	4/6/2016
KY785445	USA Florida	19/7/2016
KY785448	Honduras Francisco Morazan	10/6/2016
KY785450	Brazil Rio de Janeiro	12/4/2016
KY785452	Honduras Francisco Morazan	7/6/2016
KY785455	Brazil Rio de Janeiro	6/4/2016
KY785456	Brazil Rio de Janeiro	15/4/2016
KY785464	Puerto Rico	13/4/2016
KY785465	Dominican Republic Distrito Nacional	7/4/2016
KY785468	USA Miami Florida	5/10/2016
KY785469	Colombia Santander	5/4/2016
KY785472	USA Miami Florida	20/9/2016
KY785475	Dominican Republic Distrito Nacional	7/4/2016
KY785476	Dominican Republic Distrito Nacional	18/4/2016
KY785484	Dominican Republic	6/6/2016
KY921911	Singapore	1/6/2016
KY927808	China Henan	15/9/2016
KY989971	Colombia	15/12/2015
LC171327	Japan	20/7/2016
LC190723	JapanKanagawa Yokohama	20/5/2016
LC191864	JapanChiba	21/4/2016
LC219720	Japan	22/11/2016
LC331561	Japan Tokyo	1/6/2016
LC369584	Japankanagawa Sagamihara	1/6/2017
MF03611	China	2/11/2016
MF07335	Brazil	1/2/2016
MF07335	Brazil	1/6/2015
MF07335	Brazil	1/3/2015
MF09876	Russia	31/5/2016
MF09876	Russia	25/8/2016
MF09877	Russia	30/1/2017
MF09965	China Guizhou	08/2016/15
MF15953	USA	19/4/2017
MF16736	China	26/2/2016
MF17341	India	14/11/2016
MF35214	Brazil Pernambuco	13/5/2015
MF38432	Haiti	17/5/2016

MF43451(	Nicaragua Managua	5/8/2016
MF43451	Nicaragua Managua	19/8/2016
MF43452:	Nicaragua Managua	19/7/2016
MF43452:	Nicaragua Managua	29/8/2016
MF43828(	Cuba	12/2/2017
MF51085	Senegal	12/6/1984
MF57455:	Colombia Barranquilla	15/12/2015
MF57455!	Colombia Barranquilla	15/12/2015
MF57456!	Colombia Barranquilla	15/12/2015
MF57456(	Colombia Barranquilla	15/12/2015
MF57456	Colombia Barranquilla	15/12/2015
MF57457(	Colombia Barranquilla	15/12/2015
MF57457:	Colombia Barranquilla	15/12/2015
MF57457:	Colombia Barranquilla	15/12/2015
MF57457!	Colombia Barranquilla	15/12/2015
MF57457	Colombia Barranquilla	15/12/2015
MF57457!	Colombia Barranquilla	15/12/2016
MF57457!	Colombia Barranquilla	15/12/2016
MF57458:	Colombia Barranquilla	15/12/2016
MF57458	Colombia Barranquilla	15/12/2016
MF57458!	Colombia Barranquilla	15/12/2016
MF57458(	Colombia Barranquilla	15/12/2016
MF57458	Colombia Barranquilla	15/12/2016
MF57458!	Colombia Barranquilla	15/12/2016
MF62979(	Nigeria	5/4/2011
MF62979	Nigeria	5/7/2013
MF62979!	Senegal	10/7/2013
MF62979!	Senegal	11/11/2000
MF66443(	Russia Moscow	25/8/2016
MF69277!	Taiwan	15/10/2016
MF78307:	Haiti	27/6/2016
MF78307:	Haiti	10/5/2016
MF79497:	Ecuador	1/5/2016
MF80137!	Guatemala	21/7/2016
MF80138:	Honduras	1/6/2016
MF80138:	Honduras	3/6/2016
MF80138	Honduras Roatan	3/2/2016
MF80138(	Honduras Roatan	24/2/2016
MF80138	Honduras Roatan	29/3/2016
MF80138!	Honduras Roatan	10/5/2016
MF80139:	Mexico Oaxaca	6/7/2016
MF80139!	Mexico Chiapas	9/5/2016
MF80139(	Mexico Chiapas	17/5/2016
MF80139	Mexico Campeche	20/5/2016
MF80139!	Mexico Chiapas	20/5/2016
MF80140(	Mexico Chiapas	20/5/2016
MF80140:	Mexico Chiapas	23/5/2016
MF80140:	Mexico Chiapas	26/5/2016
MF80140:	Mexico Chiapas	25/5/2016
MF80140(	Mexico Oaxaca	7/7/2016
MF80140!	Mexico Chiapas	5/7/2016

MF80141(	Mexico Guerrero	5/7/2016
MF80141:	Mexico Guerrero	7/7/2016
MF80141:	Mexico Guerrero	7/7/2016
MF80141‹	Mexico Guerrero	5/7/2016
MF80141	Mexico Chiapas	1/7/2016
MF80141!	Mexico Chiapas	30/6/2016
MF80142:	Mexico Guerrero	30/6/2016
MF80142(	Nicaragua	25/7/2016
MF92650!	Nigeria	13/10/2016
MF98873‹	Singapore	15/8/2017
MF98874:	USA Miami Florida	1/9/2016
MF99680‹	Thailand	7/8/2017
MG36690	Thailand	27/6/2015
MG49469	Mexico Veracruz	24/11/2016
MG54866	Thailand	30/11/2016
MG54866	Thailand	30/11/2016
MG59521	Mexico	23/6/2016
MG64598	Thailand	28/10/2006
MG67471	China	16/4/2016
MG67471	China	16/4/2016
MG80764	Thailand	11/11/2016
MG80764	Thailand	7/9/2017
MG82739	French Polynesia	25/10/2013
MG98256	Chile Easter Island	29/4/2014
MH01329	Thailand	1/10/2017
MH05537	China	1/6/2016
MH06326	Cuba	24/9/2017
MH06326	Cuba	17/7/2017
MH06326	Cuba	13/8/2017
MH06326	Cuba	17/7/2017
MH11918	Thailand	1/12/2016
MH15720	Mexico Tapachula Chiapas	10/8/2016
MH15720	Mexico Tapachula Chiapas	22/8/2016
MH15721	Mexico Tapachula Chiapas	23/6/2016
MH15823	Cambodia	1/6/2010
MH15823	Puerto Rico	1/6/2015
MH17934	Colombia	10/12/2015
MH25560	Singapore	1/9/2016
MH36855	Cambodia	14/6/2016
MH43025	Vietnam Ho Chi Minh City	21/10/2016
MH43025	Vietnam Ho Chi Minh City	22/10/2016
MH51359	Brazil Sinop	9/12/2015
MH51359	Brazil Sinop	11/12/2015
MH51360	Brazil Sinop	11/12/2015
MH54470	Colombia	17/1/2016
MH88252	Brazil	26/4/2016
MH88252	Brazil	3/5/2016
MH88253	Brazil	10/5/2016
MH88253	Brazil	17/5/2016
MH88253	Brazil	24/5/2016
MH88253	Brazil	3/6/2016

MH88253	Brazil	8/6/2016
MH88253	Brazil	16/6/2016
MH88253	Brazil	28/6/2016
MH88253	Brazil	5/7/2016
MH88253	Brazil	12/7/2016
MH88253	Brazil	19/7/2016
MH88254	Brazil	27/7/2016
MH88254	Brazil	19/4/2016
MH88254	Brazil	19/4/2016
MH88254	Brazil	5/5/2016
MH88254	Brazil	12/5/2016
MH88254	Brazil	24/5/2016
MH88254	Brazil	2/6/2016
MH88254	Brazil	9/6/2016
MH90022	Mexico Morelos	1/6/2016
MK23803	India Jaipur City Rajasthan state	15/10/2018
MK24141	Cape Verde	3/12/2015
MK24141	Cape Verde	27/11/2015
MK24141	Cape Verde	4/6/2016
MK82915	Angola	25/5/2017
MK82915	Angola	15/6/2017
