# Supplementary material for: Comprehensive phylogenomic analysis of Zika virus: Insights into its origin, past evolutionary dynamics, and global spread
Source: Virus Res. 2024 Nov 8;350:199490. doi: 10.1016/j.virusres.2024.199490 (PMC11583807; doi:10.1016/j.virusres.2024.199490)
Supplement: Supplementary file 3 [file mmc3.pdf]

Supplementary Table 2

Accession	Collection	Geo	Length	Publications	Release	Usable
HQ234498	1947	Uganda	10269	22389730	19/03/2012	no, too many passages
HQ234500	1968	Nigeria	10251	22389730	20/03/2012	no, too many passages
HQ234501	1984	Senegal	10269	22389730	21/03/2012	no, too many passages
KF268948	1976	Central African Republic	10788	25514122	22/03/2012	no, too many passages; not clear
KF270886	2007	Gabon	841	24516683	23/03/2012	yes
KF270887	2007	Gabon	772	24516683	24/03/2012	yes
KF383015	2001	Senegal	753	24421913	25/03/2012	no, too short
KF383016	2001	Senegal	753	24421913	26/03/2012	no, too short
KF383017	2001	Senegal	753	24421913	27/03/2012	no, too short
KF383018	2000	Senegal	753	24421913	28/03/2012	no, too short
KF383019	1998	Senegal	753	24421913	29/03/2012	no, too short
KF383020	1980	Cote d'Ivoire	753	24421913	30/03/2012	no, too short
KF383021	1998	Senegal	753	24421913	31/03/2012	no, too short
KF383022	1997	Senegal	753	24421913	01/04/2012	no, too short
KF383023	1997	Senegal	753	24421913	02/04/2012	no, too short
KF383024	1997	Senegal	753	24421913	03/04/2012	no, too short
KF383025	1997	Senegal	753	24421913	04/04/2012	no, too short
KF383026	1997	Senegal	753	24421913	05/04/2012	no, too short
KF383027	1997	Senegal	753	24421913	06/04/2012	no, too short
KF383028	2002	Senegal	753	24421913	07/04/2012	no, too short
KF383029	2002	Senegal	753	24421913	08/04/2012	no, too short
KF383030	1981	Burkina Faso	753	24421913	09/04/2012	no, too short
KF383031	1969	Senegal	753	24421913	10/04/2012	no, too short
KF383032	1979	Senegal	753	24421913	11/04/2012	no, too short
KF383033	1979	Senegal	753	24421913	12/04/2012	no, too short
KF383034	1979	Senegal	753	24421913	13/04/2012	no, too short
KF383035	1963	Uganda	735	24421913	14/04/2012	no, too short
KF383036	1999	Cote d'Ivoire	753	24421913	15/04/2012	no, too short
KF383037	1996	Cote d'Ivoire	753	24421913	16/04/2012	no, too short
KF383038	1999	Cote d'Ivoire	753	24421913	17/04/2012	no, too short
KF383039	1991	Senegal	753	24421913	18/04/2012	no, too short
KF383040	1990	Cote d'Ivoire	753	24421913	19/04/2012	no, too short
KF383041	1990	Cote d'Ivoire	753	24421913	20/04/2012	no, too short
KF383042	1990	Cote d'Ivoire	753	24421913	21/04/2012	no, too short
KF383043	1990	Cote d'Ivoire	753	24421913	22/04/2012	no, too short
KF383044	1990	Cote d'Ivoire	753	24421913	23/04/2012	no, too short
KF383045	1990	Cote d'Ivoire	753	24421913	24/04/2012	no, too short
KF383046	1999	Cote d'Ivoire	753	24421913	25/04/2012	no, too short
KF383084	1991	Senegal	708	24421913	26/04/2012	no, too short
KF383085	1969	Senegal	708	24421913	27/04/2012	no, too short
KF383086	1999	Cote d'Ivoire	708	24421913	28/04/2012	no, too short
KF383087	1979	Senegal	708	24421913	29/04/2012	no, too short
KF383088	1979	Senegal	708	24421913	30/04/2012	no, too short

KF383089	2002	Senegal	708	24421913	01/05/2012	no, too short
KF383090	2002	Senegal	708	24421913	02/05/2012	no, too short
KF383091	2001	Senegal	708	24421913	03/05/2012	no, too short
KF383092	2001	Senegal	708	24421913	04/05/2012	no, too short
KF383093	2001	Senegal	708	24421913	05/05/2012	no, too short
KF383094	2000	Senegal	708	24421913	06/05/2012	no, too short
KF383095	1998	Senegal	708	24421913	07/05/2012	no, too short
KF383096	1998	Senegal	708	24421913	08/05/2012	no, too short
KF383097	1997	Senegal	708	24421913	09/05/2012	no, too short
KF383098	1997	Senegal	708	24421913	10/05/2012	no, too short
KF383099	1997	Senegal	708	24421913	11/05/2012	no, too short
KF383100	1997	Senegal	708	24421913	12/05/2012	no, too short
KF383101	1997	Senegal	708	24421913	13/05/2012	no, too short
KF383102	1997	Senegal	708	24421913	14/05/2012	no, too short
KF383103	1999	Cote d'Ivoire	708	24421913	15/05/2012	no, too short
KF383104	1999	Cote d'Ivoire	708	24421913	16/05/2012	no, too short
KF383105	1996	Cote d'Ivoire	708	24421913	17/05/2012	no, too short
KF383106	1990	Cote d'Ivoire	708	24421913	18/05/2012	no, too short
KF383107	1990	Cote d'Ivoire	708	24421913	19/05/2012	no, too short
KF383108	1990	Cote d'Ivoire	708	24421913	20/05/2012	no, too short
KF383109	1981	Burkina Faso	708	24421913	21/05/2012	no, too short
KF383110	1990	Cote d'Ivoire	708	24421913	22/05/2012	no, too short
KF383111	1990	Cote d'Ivoire	708	24421913	23/05/2012	no, too short
KF383112	1990	Cote d'Ivoire	708	24421913	24/05/2012	no, too short
KF383113	1980	Cote d'Ivoire	708	24421913	25/05/2012	no, too short
KF383114	1979	Senegal	708	24421913	26/05/2012	no, too short
KU720415	1947	Uganda	10766		27/05/2012	no, too many passages
KU955591	1984-11-20	Senegal	10806	27174284	28/05/2012	yes, 3 passages,
KU955592	1984-12-06	Senegal	10806	27174284	29/05/2012	yes, 3 passages,
KU955594	1947-04	Uganda	10795	27174284	30/05/2012	no, too many passages
KU955595	1984-12-14	Senegal	10806	27174284	31/05/2012	yes,3 passage,
KX377335	1947-04	Uganda	10807		01/06/2012	no, too many passages
KX421193	1947	Uganda	10269	27443522	02/06/2012	no, too many passages
KX601166	1984-11-17	Senegal: Kedougou	10771		03/06/2012	Do not know
KX601169	1947-04-20	Uganda: Entebbe	10648		04/06/2012	no, too many passages
KY288905	1962-11	Uganda	10752		05/06/2012	unknow history, NCPV:1308258v
KY576904	1989	Central African Republic: Bangui	1358		06/06/2012	no, unknown cell passages
KY989511	1947	Uganda	10807		07/06/2012	no, too many passages
MF510857	1984-06-12	Senegal	10802		08/06/2012	yes
MF629796	2011-04-05	Nigeria	1482	28398562	09/06/2012	yes
MF629797	2013-07-05	Nigeria	1427	28398562	10/06/2012	yes
MF629798	2013-07-10	Senegal	1512	28398562	11/06/2012	yes
MF629799	2000-11-11	Senegal	1390	28398562	12/06/2012	yes
MF926508	2016-10-13	Nigeria	841	29885620	13/06/2012	yes
MK028860	1984	Senegal: Kedougou	10771		14/06/2012	no
MK241415	2015-12-03	Cape Verde: Santiago	10617		15/06/2012	yes

MK241416	2015-11-27	Cape Verde: Fogo	10617		16/06/2012	yes
MK241417	2016-06-04	Cape Verde: Fogo	10617		17/06/2012	yes
MK829152	2017-05-25	Angola: Bengo, Caxito	10164	31559967	18/06/2012	yes
